# Vitamin D Supplementation in Central Nervous System Demyelinating Disease—Enough Is Enough

**DOI:** 10.3390/ijms20010218

**Published:** 2019-01-08

**Authors:** Darius Häusler, Martin S. Weber

**Affiliations:** 1 Institute of Neuropathology, University Medical Center, 37099 Göttingen, Germany; darius.haeusler@med.uni-goettingen.de; 2 Department of Neurology, University Medical Center, 37099 Göttingen, Germany

**Keywords:** multiple sclerosis, vitamin D, vitamin D receptor, experimental autoimmune encephalomyelitis, T cells, hypercalcemia

## Abstract

The exact cause of multiple sclerosis (MS) remains elusive. Various factors, however, have been identified that increase an individual’s risk of developing this central nervous system (CNS) demyelinating disease and are associated with an acceleration in disease severity. Besides genetic determinants, environmental factors are now established that influence MS, which is of enormous interest, as some of these contributing factors are relatively easy to change. In this regard, a low vitamin D status is associated with an elevated relapse frequency and worsened disease course in patients with MS. The most important question, however, is whether this association is causal or related. That supplementing vitamin D in MS is of direct therapeutic benefit, is still a matter of debate. In this manuscript, we first review the potentially immune modulating mechanisms of vitamin D, followed by a summary of current and ongoing clinical trials intended to assess whether vitamin D supplementation positively influences the outcome of MS. Furthermore, we provide emerging evidence that excessive vitamin D treatment via the T cell-stimulating effect of secondary hypercalcemia, could have negative effects in CNS demyelinating disease. This jointly merges into the balancing concept of a therapeutic window of vitamin D in MS.

## 1. Introduction

**Figure 1:**
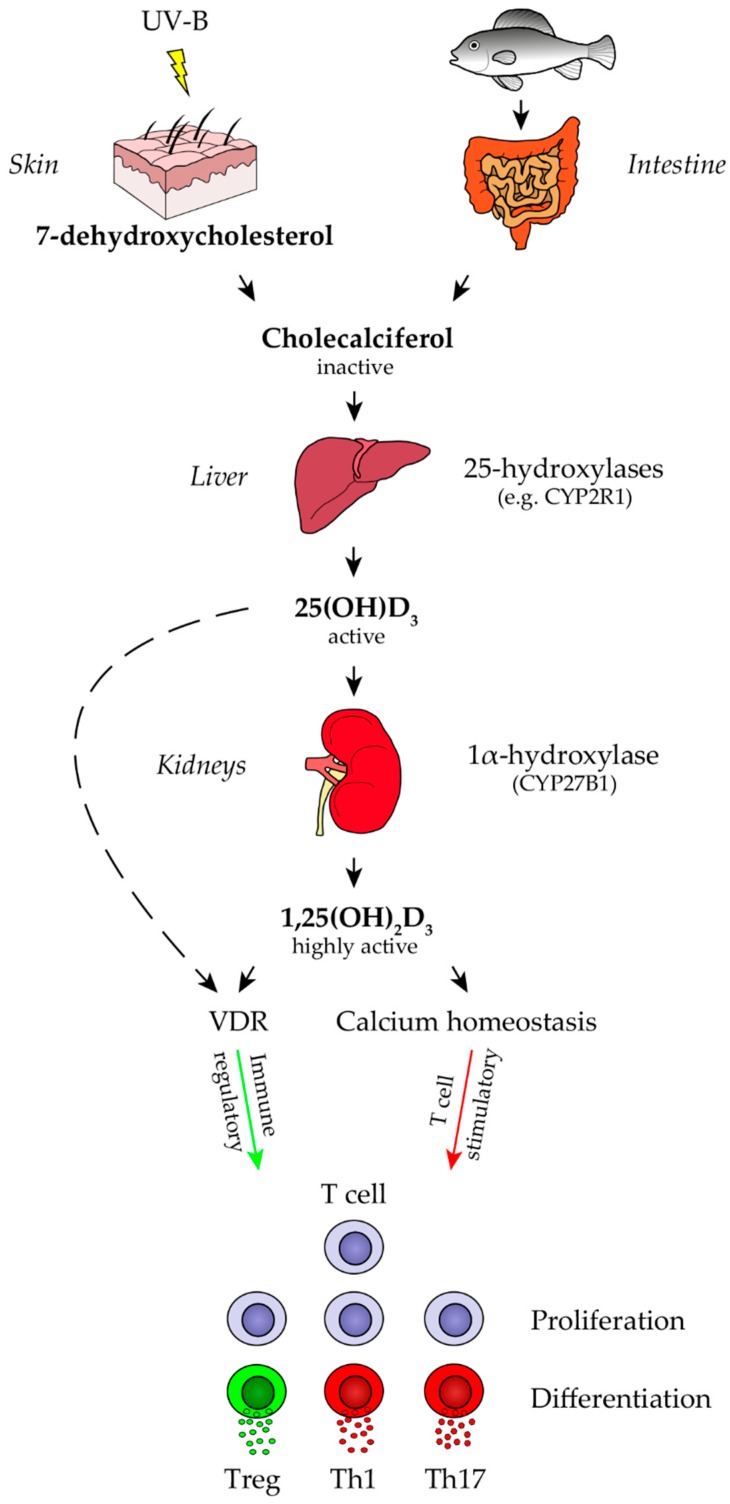
Vitamin D metabolism. Vitamin D (cholecalciferol) can be obtained from dietary intake or by synthesis in the skin from 7-dehydroxycholesterol in response to ultraviolet (UV) light. The first step in vitamin D metabolism occurs in the liver, where it is hydroxylated by 25-hydroxylases (CYP2R1, CYP27A1 and CYP3A4) towards 25-hydroxyvitamin D (25(OH)D_3_). The second step in vitamin D metabolism takes place mainly in the kidneys, where it is hydroxylated by 1α-hydroxylase (CYP27B1) to the biologically most active form of vitamin D, 1,25-dihydroxyvitamin D_3_ (1,25(OH)_2_D_3_). Both 1,25(OH)_2_D_3_ and 25(OH)D_3_ are immune modulatory upon binding to a vitamin D receptor (VDR) present in the nucleus of almost all immune cells. However, 25(OH)D_3_ shows a 100-fold less binding affinity as compared to 1,25(OH)_2_D_3_. Other functions of 1,25(OH)_2_D_3_ is the regulation of intestinal calcium and phosphate absorption, calcium mobilization from bone and reabsorption of calcium in the kidney. Secondary hypercalcemia, mediated by high serum vitamin D levels, may lead to a T cell stimulatory effect.

Various factors have been discovered which determine an individual’s risk of developing multiple sclerosis (MS), a chronic demyelinating disease of the central nervous system (CNS). Earlier family studies, as well as later molecular analyses, suggest a genetic predisposition for development of MS. Intriguingly, the vast majority of all genetic risk factors identified to date, encode for parts of the immune system. This supports the concept that an overwhelming (auto-)immune response leads to CNS inflammation, demyelination and neurodegeneration in the pathogenesis and progression of MS. Besides these risk genes, some environmental factors have been suggested to be involved in triggering and perpetuating MS pathogenesis [1,2]. Infections, such as a symptomatic Epstein Barr virus (EBV) at a vulnerable age [3], inhalative smoking [4] as well as lack of sun light exposure [5] and low levels of vitamin D [6] have been reported to enhance the risk of developing MS. The latter two factors could be interdependent, as the primary form of vitamin D, cholecalciferol (vitamin D3) is generated in the skin upon ultraviolet (UV) radiation. Alternatively, vitamin D can be taken up with food, such as dark fish (Figure 1). While diet is considered the minor source of vitamin D [7], it may become essential when UVB exposure is increasingly restricted, both by active prevention, as well as environmental changes [8,9,10]. Regardless of its relative contribution under physiological conditions, vitamin D levels can be effectively and rapidly raised by diet. Accordingly, the possible association between vitamin D and MS, and so the intuitively easy elimination of a potential MS risk factor by vitamin D supplementation has gained much interest over recent years.

## 2. Vitamin D and Multiple Sclerosis

Several aspects suggest that low levels of vitamin D could contribute to MS in a pathogenic manner. Firstly, MS patients generally have relatively low levels of vitamin D, which further decline throughout their disease course [11,12]. Most importantly, low serum levels of vitamin D are associated with an enhanced risk of developing de novo MS when analyzed in a large cohort of individuals prospectively [13]. In patients with established MS, vitamin D levels above 70 nmol/L were found to be associated with a decreased risk of attacks [14]. In contrast, lower concentrations increased the likelihood of both relapses and early chronic progression [15,16]. Further supporting a direct effect of vitamin D in MS, genome-wide association studies have identified that genetic abnormalities in genes encoding 1α-hydroxylase, the rate-limiting enzyme for the conversion of vitamin D into its active metabolite, increased the risk of developing MS [17]. It can thus be concluded, that a higher serum level of functional vitamin D is associated with a reduced MS risk and severity. However, a causal protective effect and accordingly, whether therapeutically raising the vitamin D level alters disease severity, is a matter of ongoing debate.

### 2.1. Vitamin D Metabolism and Direct Effects of Vitamin D Metabolites

To assess a potential clinical benefit of vitamin D in MS and other autoimmune conditions, it is instrumental to recall the metabolism of this vitamin in humans. Vitamin D (cholecalciferol) is a secosteroid hormone that can be obtained from dietary intake or by synthesis in the skin from 7-dehydroxycholesterol in response to UV light (Figure 1) [18,19,20]. In the circulation, vitamin D binds to a specialized carrier protein, vitamin D-binding protein (DBP) and is transported to the liver, where it is hydroxylated by 25-hydroxylases (CYP2R1, CYP27A1 and CYP3A4) to 25-hydroxyvitamin D (25(OH)D_3_). 25(OH)D_3_ is the most abundant metabolite in the circulation, with a half-life of 20–90 days [21,22]. Primarily based on its relative stability, measurement of this metabolite is the most accepted parameter to assess the overall vitamin D status in an individual patient. The second step in vitamin D metabolism takes place mainly in the kidney. Here, it is hydroxylated by 1α-hydroxylase (CYP27B1) to the biologically most active form of vitamin D, 1,25-dihydroxyvitamin D_3_ (1,25(OH)_2_D_3_) [19,23]. The conversion into 1,25(OH)_2_D_3_ can also be performed by several immune cells, which also express CYP27B1, such as macrophages, monocytes, dendritic cells (DC), B cells and T cells [24,25]. Most biologic functions of 1,25(OH)_2_D_3_ are mediated by its strong and specific binding to a vitamin D receptor (VDR) present in the nucleus of almost all immune cells, including T cells [26], dendritic cells [23], monocytes and macrophages [26,27], activated B cells [25], as well as neuronal and glial cells [28,29,30,31,32,33,34,35,36]. 25(OH)D_3_ is also able to bind to VDR with a 100-fold less binding affinity as compared to 1,25(OH)_2_D_3_ [37]. Upon binding, a chain of genomic events is triggered, resulting in the transcriptional control of vitamin D-regulated genes [38,39,40]. After completion, 1,25(OH)_2_D_3_ is catabolized by repeated oxidations, converted to calcitroic acid and then excreted [37].

Most of the studies providing insight into how 1,25(OH)_2_D_3_ modulates the immune system were done in vitro. Monocytes cultured in the presence of 1,25(OH)_2_D_3_ showed a VDR-dependent loss of MHCII [41,42] and a reduction in co-stimulatory molecules, such as CD40, CD80 and CD86 [43]. This resulted in a diminished capability to induce proliferation of T cells upon stimulation with tetanus toxoid or CD40L [41,43]. Moreover, 1,25(OH)_2_D_3_ exposure to monocytes decreased the secretion of IL-1α, IL-6, IL-12, TNF-α and IP-10 [44,45,46,47], increased the transcription levels of IL-10 RNA [42,48] and enhanced their phagocytic function [42]. Monocytes can differentiate into immature DC in the presence of GM-CSF and IL-4 [49,50] and can be further differentiated into mature DC upon TNF-α, LPS, IL-1 or CD40L incubation in vitro [45,51,52]. Exposure to 1,25(OH)_2_D_3_ restored their monocytic phenotype [52] and inhibited DC maturation [51,52,53,54]. Furthermore, differentiated mature DCs, in the presence of 1,25(OH)_2_D_3,_ showed a downregulation of CD40, CD80, CD86, as well as MHCII. They also showed a diminished release of Th1 and Th17 cell-inducing cytokines, such as IL-12 and IL-23, while the production of IL-10 and CCL22, involved in Treg and Th2 immune responses, was enhanced respectively [51,52,53]. In addition, co-culture of mature DC with T cells in the presence of 1,25(OH)_2_D_3_, resulted in less T cell proliferation [51,52,54]. A direct effect of 1,25(OH)_2_D_3_ on microglial cells and antigen presenting cells (APC) within the CNS, included an inhibition of TNF-α, IL-6 and Nitric Oxide production in vitro [55]. B cells express negligible amounts of VDR and have been shown to be a target of 1,25(OH)_2_D_3_ after activation, due to their VDR up-regulation [56,57]. Nevertheless, exposing B cells to 1,25(OH)_2_D_3_ inhibited their proliferation [25,58,59], plasma cell differentiation including immunoglobulin G and -M secretion, memory B cell generation and induced B cell apoptosis in proliferating B cells [25]. T cell subsets show a high variability in their VDR expression levels. In contrast to high VDR levels on cytotoxic CD8^+^ T cells, Th2-, Th17- and Treg cells, Th1 cells exhibit moderate VDR expression, which is increased upon activation [26,60,61,62]. Exposing T cells to 1,25(OH)_2_D_3_ inhibited T cell proliferation, as well as Th1- and Th17-derived cytokine production, such as IL-2 [63,64,65,66], IFN-γ, IL-17, IL-21 and IL-22 [62,64,67,68,69]. However, it promoted the release of Th2- and Treg-derived cytokines, including IL-4, IL-5 [70] and IL-10 [71,72,73]. Moreover, 1,25(OH)_2_D_3_ induced cell-cycle arrest and apoptosis of activated effector T cells mediated by the Fas/FasL system or IL-2 [74,75,76]. Taken together, vitamin D and its metabolites clearly alter phenotype and function of various immune cells in vitro, and exert the vast majority of these immune modulatory properties via interaction with the VDR.

### 2.2. Vitamin D Supplementation Studies in MS

In light of this strong in vitro evidence, and the fact that a low vitamin D level is a negative predictor in MS, it is intuitive to study its supplementation in MS. Unfortunately, the question whether vitamin D exerts a therapeutic effect in established CNS demyelinating disease is much less clear than the in vitro evidence. In part, this is likely due to the complex interaction of vitamin D and its metabolites, not only with immune cells, but various other tissues and organs affecting regulation of multiple hormones and homeostasis of ions (Figure 1). A direct effect of vitamin D on MS activity is the requirement for all current efforts to therapeutically raise its level in affected patients. In the most widely used preclinical model of MS, murine experimental autoimmune encephalomyelitis (EAE), vitamin D indeed appeared to prevent its development [77] and to reversibly block EAE progression [78]. This effect was associated with an impaired activation and CNS migration of monocytes [79,80] and T cells [81], as well as an accentuation of anti-inflammatory natural killer T cell properties [82]. In MS patients, moderate vitamin D supplementation increased the serum level of transforming growth factor beta (TGF-β) [63] and reduced the frequency of Th17- and effector memory T cells [83]. Empirical vitamin D supplementation studies have so far provided conflicting results [84,85], and failed to conclusively establish such causality. For example, adding vitamin D_3_ to interferon beta (IFN-β) treatment, reduced MRI activity in a small trial with relapsing-remitting MS patients [86]. In this context, a study suggested that both components interact to modulate MS disease activity. This is in the sense that, an elevated level of vitamin D is a pre-requisite for IFN-β to properly function, and not necessarily a beneficial factor by itself [87]. Another study indicated that the initiation of IFN-β treatment was associated with a significant reduction in all MRI outcomes, irrespective of the patients’ vitamin D level. This suggests that the anti-inflammatory effect of increasing vitamin D levels is small when compared to IFN-β treatment. Moreover, it is controversial whether genetic variation in Wilms’ tumor gene product 1 (WT1) plays any role in regulating the relationship between IFN-β and serum 25-hydroxyvitamin D [88,89]. Similarly, in MS patients treated with natalizumab, supplementing vitamin D was reported to reduce the relapse rate when compared to its frequency prior to vitamin D supplementation [90]. Interpretation of this result, however, may be hampered by an increase in the efficacy of natalizumab itself over time [91], as the study was not controlled by a group in which vitamin D levels remained low. In the first trial examining the safety of high oral vitamin D treatment, 25 patients received escalating doses ranging from 4000 IU up to 40,000 IU per day for 28 weeks. This was followed by another 28 weeks, in which the patients were down-titrated to 4000 IU per day [92]. According to the authors, high-dose vitamin D administration was well tolerated. That being said, a follow-up study evaluating the clinical effect of high-dose vitamin D treatment in patients with MS, failed to show a significant clinical benefit of the high over the low-dose group [84]. In the largest interventional study thus far (SOLAR study), 229 participants were randomized to receive either daily oral cholecalciferol (14,000 IU vitamin D3) or placebo as add-on therapy to 44-µg IFN-β. Vitamin D supplementation significantly reduced the number of new MRI lesions in patients receiving IFN-β. However, the ambitious primary endpoint of the study, being a change in the proportion of patients with “no evidence of disease activity”, was not formally reached [93,94]. Larger ongoing trials aim to determine more conclusively whether interventional vitamin D supplementation directly alters relapse frequency or accumulating disability, independent of any respective co-medication in MS [95,96].

In light of these heterogeneous results thus far, alternative concepts corroborating the correlation of sunlight and MS disease activity currently arise. Exposure of the skin to sun has numerous biological effects, with UV radiation probably exerting the greatest impact. In this regard, a recent report highlights that higher actinic skin damage, as a result of UV exposure, is associated with a reduction in MS inflammatory events, independent of concomitantly raised vitamin D levels [97]. Besides promoting vitamin D synthesis, UV-B is absorbed to a large extent by urocanic acid (UCA), hereby converting from the trans-isomer to its active form cis-UCA, which conveys both cutaneous and systemic immunosuppression [98]. Cis-UCA plasma levels were found to be lower in RR-MS patients compared to healthy controls [99], this parallels vitamin D’s properties [100,101]. Cis-UCA in vitro reduces the pro-inflammatory antigen presenting capacity of myeloid APCs and promotes development of Treg [99]. Enhanced Treg function and frequency is probably the most consistent immunoregulatory effect to occur upon UV irradiation. A recent study mechanistically dissected this concept in mice. UV-B light facilitated development of tolerogenic DC, which in return fostered development of EAE-ameliorating Treg [102]. Most importantly, this immune-mediated clinical effect occurred in the absence of a detectable increase in 25-(OH)D_3_ [103]. Together with the parallel observation that mice with genetically disrupted vitamin D signaling are fully susceptible to UV-mediated immunosuppression [104,105], these findings substantially question whether vitamin D is the central metabolite in mediating the beneficial effect of UV irradiation in CNS demyelinating disease and in explaining the association of MS with higher latitude [106].

### 2.3. Possible Side Effects of Secondary Hypercalcemia

As mentioned above and illustrated in Figure 1, vitamin D and its metabolites regulate multiple hormones and homeostasis of ions. Specifically, 1,25(OH)_2_D_3_ influences the secretion of hormones such as prolactin, parathyroid hormone (PTH), as well as insulin [37] and has complex functions in calcium and phosphorus homeostasis. The latter includes regulation of intestinal calcium and phosphate absorption, calcium mobilization from bone and reabsorption of calcium in the kidney [107]. Elevated serum 1,25(OH)_2_D_3_ in humans, hereby leads to an increased uptake of calcium from the intestines, resorption in the kidneys and may result in secondary hypercalcemia [108]. This is especially true when combined with calcium intake [109,110,111].

Calcium ions (Ca2^+^) are essential second messengers in the human body and their widespread roles in biology are mirrored in the immune system [112]. Engagement of several different tyrosine and non-tyrosine kinase receptors stimulate Ca2^+^ influx in immune cells, including T cells, B cells and monocytes/macrophages [113]. In T cells, antigen engagement of the T cell receptor triggers Ca2^+^ release from intracellular stores in the endoplasmic reticulum (ER). This transient Ca2^+^ release opens calcium-release activated channels (CRAC) in the plasma membrane. This, in conjunction with voltage-gated calcium channels (VGCC), results in a massive influx of extracellular Ca2^+^ into the cytosol followed by activation of calcineurin and nuclear import of NFATc proteins. These regulate immune-response genes, encoding for cell proliferation, differentiation, migration and production of cytokines [114]. Several studies suggest that extracellular calcium influx, via voltage-gated calcium channels contributes to white matter damage in acute spinal cord injury and stroke. In experimental autoimmune encephalomyelitis (EAE), the animal model of MS, administration of calcium channel blockers ameliorated disease [115], decreased microglial proinflammatory activity [116], fostered remyelination and induced microglia-specific apoptosis [117]. Moreover, a diminished calcium activity caused by EDTA injections showed a therapeutic effect in EAE [118]. While there is currently no molecule to selectively interfere with calcium influx into immune cells in vivo, genetically engineered T cells with non-functioning calcium channels completely blocked EAE by preventing development of myelin-reactive Th1 and Th17 cells [119,120]. In this context, we recently revisited the effect of two different doses of vitamin D in EAE [121]. We fed mice with cholecalciferol, the metabolite most commonly supplemented in humans. We measured the serum level of 25(OH) vitamin D, which used in humans to assess the vitamin D status after several weeks. In our three dosing groups we set levels reflective of MS patients who are (a) vitamin D-deficient, (b) modestly supplemented and (c) treated in MS high dose supplementation trials [92,122]. As consistently observed in other studies, moderately substituting a low vitamin D status ameliorated EAE, which is normally associated with a dampened development and expansion of encephalitogenic T cells. In sharp contrast, we detected that supplementation with high doses of vitamin D exerted an activation of innate and adaptive immune cells, associated with enhanced CNS immune infiltration and a distinctive acceleration of EAE severity. Importantly, these mice containing vitamin D serum levels exactly mirroring the ones in humans on high dose supplementation, showed a subtle but significant rise in their mean serum calcium. Modelling these calcium levels in vitro revealed that T cell activation was strongly enhanced in a culture medium with higher calcium content. Importantly, this was the case when murine T cells were used, but even more so when human T cells were examined. Upon activation, an enhanced calcium influx triggered proliferation and pro-inflammatory differentiation of T cells. This corroborates a causal sequence of high dose vitamin D treatment, secondary hypercalcemia and a promoted development of disease-driving encephalitogenic T cells.

Importantly, hypercalcemia to the extent comparable to the levels associated with deterioration of EAE, commonly occurs in humans supplemented with high doses of vitamin D [108]. This is especially true when combined with calcium intake [109,110,111]. High dose vitamin D was also reported to cause hypercalcemia in the treatment of MS [123], which was associated with development of severely disabling relapses as well as increased MRI activity [124]. Along the same lines, recent clinical trials revealed enhanced immune cell activation in MS patients supplemented with 50,000 IU of vitamin D_3_ every five days [125]. Whereas the functionally opposite outcome occurred at moderate vitamin D levels [83,94]. In conjunction with our pre-clinical observation, these findings may indicate that the immunological and clinical benefit of vitamin D is reflective of immune-regulatory vitamin D receptor signaling. However, these desirable effects are abolished by a secondary rise in the mean calcium level when a certain dose threshold of vitamin D is exceeded. Therefore, we recommend a vitamin D serum level between 75–125 nmol/L, as proposed by other clinicians [126]. This range of vitamin D has been associated with low risk of developing MS and low disease activity and can be easily reached with adequate sun exposure and vitamin D balanced diet without any additional vitamin D supplementation.

## 3. Conclusions

There is little doubt that a low vitamin D status is a risk factor for development and progression of MS. In part, this may reflect a true deficit in vitamin D itself, on the other hand, low vitamin D levels may be indicative of a lack of sun exposure, which appears to mediate beneficial effects independent or in addition to raising the vitamin D levels. Although, controlled supplementation studies in patients with MS suggest that therapeutically raising vitamin D in affected patients may positively influence the course of disease, conclusive evidence is unfortunately still lacking. Emerging studies caution that higher dose vitamin D supplementation may have the opposite clinical effect via secondary hypercalcemia having a T cell-stimulating effect. This novel concept of a relatively narrow therapeutic window for vitamin D, may also shed light on the question of why clinical trials often using higher doses of vitamin D failed or yielded conflicting results. In conclusion, vitamin D should be supplemented at moderate doses in a serum level-controlled manner. Patients should be also assessed for hypercalcemia, which should be strictly avoided. In the big picture, moderate sun exposure, combined with a diverse diet including vitamin D precursors, in conjunction with a regular assessment of vitamin D serum levels, might be the best balanced and advisable strategy for patients with MS.
